# Database and Statistical Analyses of Transcription Factor Binding Sites in the Non-Coding Control Region of JC Virus

**DOI:** 10.3390/v13112314

**Published:** 2021-11-19

**Authors:** Kazuo Nakamichi, Toshio Shimokawa

**Affiliations:** 1 Department of Virology 1, National Institute of Infectious Diseases, Tokyo 162-8640, Japan; 2 Department of Medical Data Science, Graduate School of Medicine, Wakayama Medical University, Wakayama 641-8509, Japan; shimokaw@wakayama-med.ac.jp

**Keywords:** database, JC virus, non-coding control region, mutational pattern, transcription factor binding sites, statistical analysis

## Abstract

JC virus (JCV), as an archetype, establishes a lifelong latent or persistent infection in many healthy individuals. In immunocompromised patients, prototype JCV with variable mutations in the non-coding control region (NCCR) causes progressive multifocal leukoencephalopathy (PML), a severe demyelinating disease. This study was conducted to create a database of NCCR sequences annotated with transcription factor binding sites (TFBSs) and statistically analyze the mutational pattern of the JCV NCCR. JCV NCCRs were extracted from >1000 sequences registered in GenBank, and TFBSs within each NCCR were identified by computer simulation, followed by examination of their prevalence, multiplicity, and location by statistical analyses. In the NCCRs of the prototype JCV, the limited types of TFBSs, which are mainly present in regions D through F of archetype JCV, were significantly reduced. By contrast, modeling count data revealed that several TFBSs located in regions C and E tended to overlap in the prototype NCCRs. Based on data from the BioGPS database, genes encoding transcription factors that bind to these TFBSs were expressed not only in the brain but also in the peripheral sites. The database and NCCR patterns obtained in this study could be a suitable platform for analyzing JCV mutations and pathogenicity.

## 1. Introduction

The JC virus (JCV) belongs to the family Polyomaviridae and has a circular double-stranded DNA genome. JCV is widely distributed in the human population, with 60% to 80% of adults serologically positive for this virus [1,2,3,4,5]. The first infection with JCV occurs mainly in childhood, after which the virus establishes a persistent asymptomatic infection in the kidney and urinary tract [6,7]. Additionally, JCV persists or is latent in other sites, such as lymphoid tissue and bone marrow [1,8,9,10,11]. This particular JCV presents a stable genomic structure and sequence and is referred to as the archetype [12,13]. Archetype JCV is detected in the urine of 7% to 46% of immunocompetent individuals [4,14,15,16,17,18,19,20,21,22,23,24,25,26,27].

In immunocompromised patients or those treated with agents that affect cellular immunity, JCV can reactivate and cause progressive multifocal leukoencephalopathy (PML) due to lytic infection of oligodendrocytes and fatal demyelination in the brain [28,29,30,31,32]. JCV isolates from the brain and cerebrospinal fluid (CSF) of PML patients shows hypervariable mutations in the non-coding control region (NCCR; also referred to as the regulatory or transcription control regions) of the viral genome, and these variants are termed prototypes [8,28,33,34,35,36]. The nucleotide sequence of the NCCR includes transcription factor binding sites (TFBSs) and is responsible for the expression of viral early and late genes [8,28,37,38]. Rearrangement in the NCCRs of prototype JCV is thought to be generated by deletions and/or duplications in the archetypal sequences [12,39], which alter promoter activity [40,41].

In previous studies, we observed patient-specific mutation patterns in the NCCR sequences of prototype JCV derived from CSF specimens of PML cases [42,43] and similar to those reported in other investigations [10,34,36,40,44,45,46,47,48,49,50,51,52,53,54,55]. Furthermore, the mutation pattern of the most frequently detected JCV NCCRs in each patient changed during disease progression [42]. However, because of the limited number of JCV isolates obtained by a single research group and the highly complex variations, it was not feasible to distinguish apparent features in the NCCRs of prototype JCV by schematically comparing the sequences, except for the deletion occurring mainly in region D [42,43]. The nucleotide sequences of JCV NCCRs have been determined mainly in anthropological or epidemiological studies of archetype viruses and analyses of the pathogenic mechanism of prototype viruses by many researchers, including the authors [7,10,12,13,34,35,36,37,38,39,40,42,43,44,45,46,47,48,49,50,51,52,53,54,55]. To better understand the diversity and regularity of NCCR rearrangement, a platform for compiling these sequences is necessary.

Additionally, to analyze the NCCRs of polyomaviruses, including JCV, several research groups have used computer simulations to identify the types and locations of TFBSs [56,57,58,59,60,61]. Using this technique might enable the comprehensive estimation of TFBSs present in the NCCR sequences of a large variety of JCVs and analysis of these data to reveal mutation patterns. In this study, we created an annotation database of the NCCR sequences of JCV isolates registered in GenBank and examined the characteristics of TFBSs using statistical methods.

## 2. Materials and Methods

### 2.1. Acquisition of Sequence Data for JCV NCCRs

**Figure 1:**
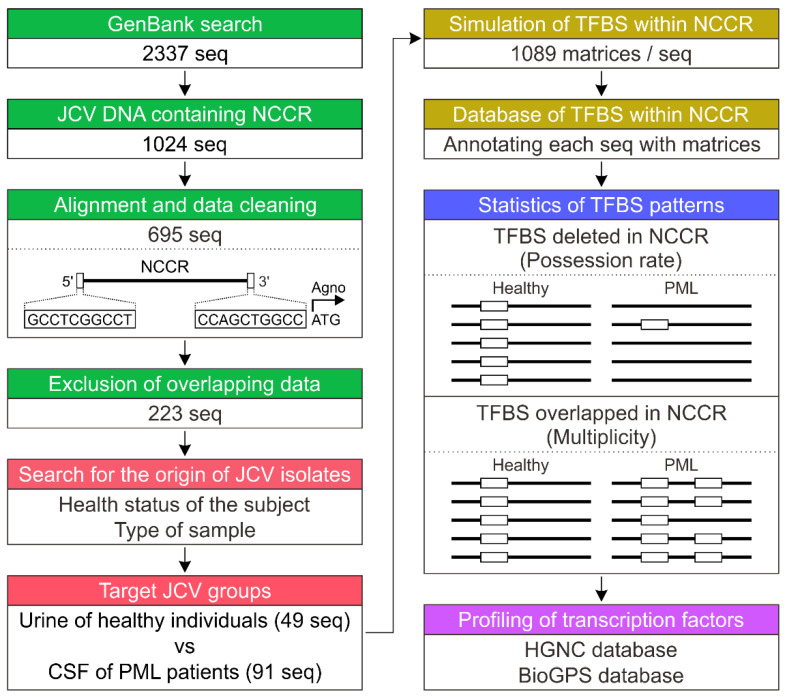
Overall workflow of the data processing and analysis of transcription factor binding sites (TFBSs) in the non-coding control region (NCCR) of JC virus (JCV). The nucleotide sequences (seq) of JCV NCCRs in GenBank were extracted and aligned, and their origins were confirmed. TFBSs in the NCCRs of JCV isolates from the urine of healthy individuals and cerebrospinal fluid (CSF) of PML patients were identified using computer simulation. A database was created based on the NCCR sequences and TFBS annotations, and the patterns and metadata of TFBSs were examined using statistical analysis and public databases.

The overall workflow of this study is summarized in Figure 1. A total of 2337 sequences of JCV DNA and their metadata were downloaded from the nucleotide database of the National Center for Biotechnology Information (NCBI; https://www.ncbi.nlm.nih.gov/, accessed date: 1 October 2013) using search terms “JC”, “polyomavirus”, and “region.” To avoid any error or bias caused by mixing data from Sanger sequencing and next-generation sequencing, DNA sequences from JCV registered over the course of 23 years (1990–2013) were used for the analysis. During this registration period, as far as we could ascertain, the DNA sequence of JCV was determined by Sanger sequencing. After importing the sequence data into the CLC Genomics Workbench software program version 7.0 (Qiagen, Aarhus, Denmark), alignments were performed using the genomic DNA sequence of the representative archetype JCV (CY strain; GenBank: AB038249.1) as a reference. The sequences were roughly aligned using 20 sequences each, and the alignment was repeated by manually checking each sequence. After this process, 1024 sequences of JCV NCCRs were obtained (Supplementary Dataset S1).

### 2.2. Data Cleaning and Extraction of JCV NCCR Sequences

The collected NCCR data were in a miscellaneous state and included partial fragments, sequences where some nucleotides had not been sequenced, or many identical sequences of NCCR clones derived from the same individuals. To obtain results with a higher degree of reliability for the computer simulation and statistical analysis of TFBSs, we cleaned the NCCR sequence data. Sequence data were extracted for a group of NCCR sequences with 5′ and 3′ nucleotide positions (1–10 and 258–267, respectively) located at both ends in the NCCR (regions A–F) of archetype JCV (CY strain) (Figure 1). Additionally, NCCR sequences containing uncertain characters other than ATGC were excluded, resulting in 695 sequences. The duplicated identical NCCR sequences were then removed by alignments, leaving 223 sequences. Review of the metadata and published literature registered in GenBank enabled examination of the types of specimens and health conditions of individuals in which each JCV sequence was detected. To accurately compare the TFBS patterns in the archetypal and prototype NCCRs, two groups of JCV NCCRs derived from the urine of healthy individuals and CSF of PML patients (49 and 91 sequences, respectively) were subjected to the following analyses as target sequences.

### 2.3. Computer Simulation of TFBSs in NCCR Sequences

TFBSs within the NCCR sequences were analyzed by computer simulation using MatInspector software (Genomatix, Munich, Germany) with Matrix Family Library version 9.2 (Genomatix). This program identifies TFBSs in nucleotide sequences using a large library of weight matrices, annotates the corresponding sites with the matrices, and presents simple metadata of TFBSs [62,63]. The matrix library used in this study included 1072 and 17 weight matrices for vertebrate TFBSs and general core promoter elements, respectively. The search parameters were configured using software defaults. The sequences processed by MatInspector were imported into the CLC Genomics Workbench as FASTA format files. Because these numerous annotations were difficult to handle owing to a combination of matrix names and their family types, each of them was manually corrected to the name of the individual matrix.

### 2.4. Statistical Analyses of TFBS Patterns

The proportions of NCCRs of JCV isolates that possessed the respective TFBSs (referred to here as “possession rates”) between groups were statistically compared using Fisher’s exact test, and the Benjamini–Hochberg method was used to adjust for multiple comparisons. Statistical significance was considered at a false discovery rate (FDR)-adjusted *Q*-value < 0.05 [64]. The multiplicity of each TFBS matrix in the NCCR of JCV isolates from healthy individuals and PML patients was analyzed using Poisson regression analysis. All analyses were conducted using R version 3.6.1 (R Foundation for Statistical Computing, Vienna, Austria). Statistical significance was set at *P* < 0.05.

### 2.5. The Gene Ontology and Expression Profiles of Transcription Factors

Transcription factors that bind to each TFBS are briefly indicated in the metadata of TFBSs presented by MatInspector. Based on this information, the ontology of the genes encoding transcription factors was searched using the Human Genome Organization Gene Nomenclature Committee (HGNC) database (https://www.genenames.org/, accessed date: 18 June 2021), and the HGNC identifiers (IDs) of these genes and their currently approved symbols and names were confirmed. The gene-expression profiles of transcription factors in human tissues or cells were retrieved by accessing the BioGPS database (http://biogps.org/, accessed date: 18 June 2021) [65] via the symbol reports of the HGNC database. The BioGPS plug-in “Gene expression/activity chart” and Affymetrix microarray dataset “GeneAtlas U133A, and gcrma” [66] were used in these data searches.

## 3. Results

### 3.1. Creation of the TFBS Database for JCV NCCR Sequences

**Figure 2:**
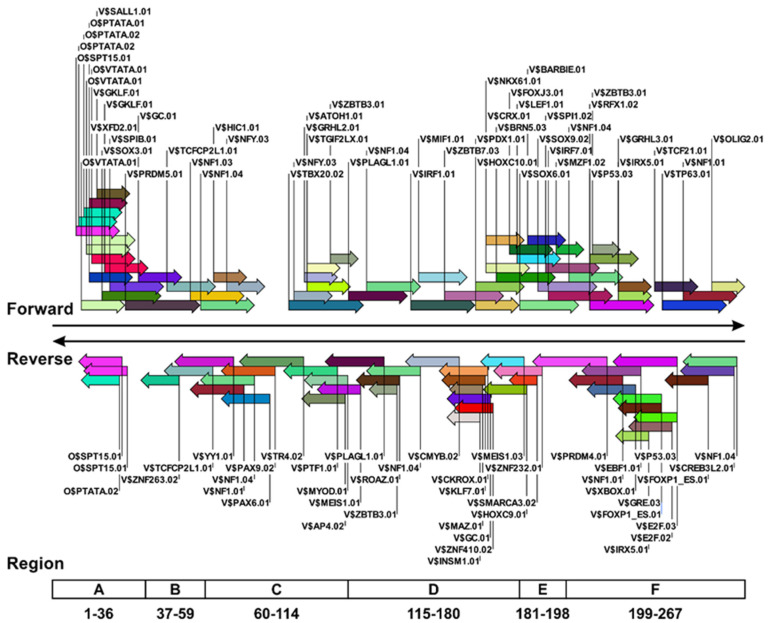
Representative example showing the type and location of TFBSs within the JCV NCCR. TFBS matrices in the NCCR sequences of 140 JCV isolates were identified using MatInspector. The data were imported into the CLC Genomics Workbench as FASTA format files, and the types and locations of TFBS matrices in each JCV isolate were annotated to NCCR sequences. Data represent all TFBS matrices identified only for the CY strain, a representative archetype JCV. The TFBS matrices (right, labeled “Forward”) were identified in the 5′ and 3′ nucleotide positions (1–267), whereas those to the left (labeled “Reverse”) were present in the complementary strand. The horizontal boxes (bottom) indicate the location and base number of regions A through F within the NCCR of the CY strain of JCV.

For the computational and statistical analyses of TFBSs according to the NCCR sequences of JCV isolates, the nucleotide sequences deposited in GenBank over a 20-year period were aligned and selected. Of the downloaded data, only ~10% (234 of 2337 sequences) comprised the full-length JCV genome, with the remainder representing partial fragments. Because a substantial number of these entries did not contain NCCRs and could not be automatically extracted as target sequences using the software, we extracted NCCRs manually by repeating the small-scale alignment using the archetype JCV genome as a reference. To simulate TFBSs more accurately in the NCCRs of various JCV isolates, the target sequences were narrowed down using 10 bases at both ends of the CY strains as landmark sequences. For the 695 NCCRs that remained after data cleaning, duplicates of precisely identical sequences were removed, and ~32% (223 sequences) remained. Most of these identical sequences were deposited during the massive sequence analysis conducted by Reid et al. [46]. Examination of the origin of the 223 extracted sequences revealed that most NCCRs were detected in the urine of healthy individuals and the CSF of PML patients (Supplementary Dataset S2). Therefore, TFBSs in the NCCRs of both groups were identified using MatInspector, and a database of TFBSs within JCV NCCR sequences was created. This database comprises a set of nucleotide sequences of each JCV isolate along with a massive number of TFBS matrices added as annotations. Moreover, the database allows the positions and sequences of any TFBS to be visualized and tabulated using standard genetic analysis software. For example, Figure 2 shows the position of the TFBSs on the NCCR of a well-known archetype JCV (CY strain). In the NCCR of the CY strain, 54 and 43 TFBS matrices were detected in the 5′ and 3′ nucleotide positions (1–267; forward) and its complementary strand (reverse), respectively. TFBS matrices were especially visible in regions A and regions D through F, but they were also found in other regions.

### 3.2. Overall View of TFBS Patterns in JCV NCCR

The patterns of TFBSs in the JCV NCCRs were observed by exporting the data from the created database. The NCCRs of 49 JCV isolates derived from the urine of healthy individuals were archetypal sequences, including those with very small insertions or deletions that could be regarded as genetic polymorphisms. The average numbers of TFBS matrices in these JCV isolates were 52.6 and 40.0 for the forward and reverse strands, respectively. The NCCRs of 91 JCV isolates detected in the CSF of PML patients showed prototypal sequences with averages of 57.1 and 38.0 TFBS matrices in the forward and reverse strands, respectively. There was no statistically significant difference in the mean total number of TFBS matrices within either strand of JCV NCCRs from the urine of healthy subjects and CSF of PML patients. These results indicated that the NCCR of JCV produces complex reconstructions in patients with PML, but that the total number of TFBSs within each NCCR is not significantly altered. Moreover, 464 types of TFBS matrices were detected in all NCCR sequences, among which there were sequences of shallow frequency and suspected to be nonspecific or biologically insignificant. Therefore, the TFBS matrices that were highly conserved in the archetypes were sorted and statistically examined to determine how they changed in the prototypes. Histogram analysis of the prevalence of each TFBS matrix in the NCCR of archetype JCV isolates showed that >95% of them had 34 and 18 matrices in the forward and reverse strands, respectively (Supplementary Figure S1). When the database was used to select and visualize each TFBS matrix in the NCCR alignments of the JCV isolates, we noticed that some matrices tended to be missing or overlapped in the prototype JCVs (Figure 1). Consequently, the following statistical analyses were conducted to analyze the patterns of TFBSs.

### 3.3. TFBSs Frequently Lost in the NCCR Sequences of Prototype JCV

**Figure 3:**
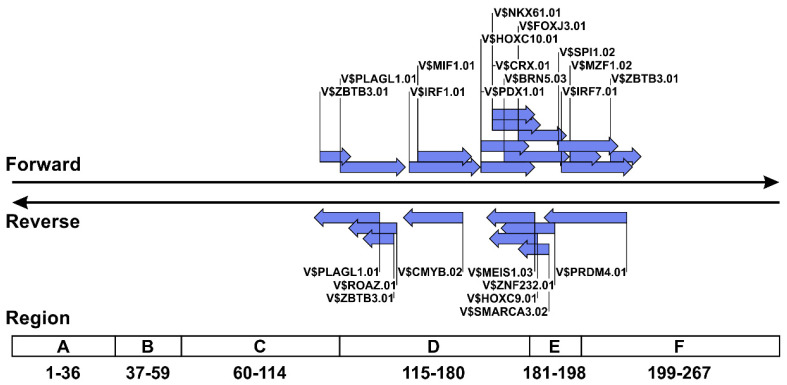
Types and locations of TFBSs lost in rearranged NCCR sequences of prototype JCV. TFBS matrices showing statistically significant decreases in possession rate in the NCCRs of prototype JCV isolates and shown in the sequence of the archetype virus (CY strain). The method of illustrating the TFBS matrices is the same as that shown in Figure 1.

Table 1 shows the TFBS matrices that showed significantly different possession rates between archetype JCVs from the urine of healthy individuals and prototype viruses from the CSF of PML patients. The NCCR sequences of prototype JCVs derived from the CSF of PML patients showed low percentages of the possession of 13 and nine matrices in the forward and reverse strands, respectively, and 15 of 22 TFBS matrices on both strands were absent in >50% of the prototypal NCCRs. Visualization of the location of these TFBSs in the NCCR of archetype JCV (CY strain) using the created annotation database revealed that the TFBS group, the possession rate of which decreased in the prototype JCVs, was mainly located in regions D through F of the NCCR of the archetype virus (Figure 3). Although the TFBS matrices identified by MatInspector are accompanied by metadata, such as the names and expression patterns of transcription factors that bind to the TFBSs, we found that this information included past designations and obscure expression sites. Therefore, the gene ontology and expression sites of transcription factors capable of binding to TFBS matrices were confirmed using the HGNC and BioGPS databases. Based on data retrieval from BioGPS using the Affymetrix microarray dataset, transcription factors that bind to TFBSs often lost in the NCCR sequences of the prototype JCVs were suggested to be expressed in various human tissues (Table 2). Additionally, some of these transcription factors were suggested to be highly expressed in sites of persistent or latent JCV infection, such as the kidney, bone marrow (CD34+ cells), and lymph nodes (Table 2). These data indicated that TFBSs, which are often lost in the NCCRs of prototype JCVs, are mainly located in regions D through F, and that the transcription factors that bind to them are expressed at a variety of peripheral sites.

### 3.4. TFBSs Likely to Multiply in the NCCR Sequences of Prototype JCVs

**Figure 4:**
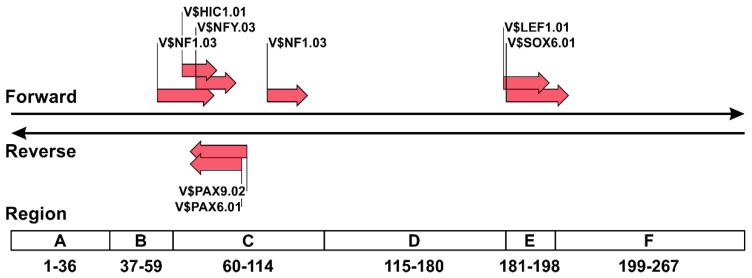
Types and locations of TFBSs tend to overlap in rearranged NCCR sequences of prototype JCV. TFBS matrices with statistically higher multiplicity in the NCCR of the prototype JCV as compared with those of the archetype JCV were determined by Poisson distribution for modeling count data. The locations of these matrices are depicted in the NCCR sequence of archetype JCV (CY strain).

We performed a final set of analyses to examine TFBSs that tend to overlap in the rearranged NCCRs of prototype JCVs using the Poisson distribution for modeling count data. Only seven of 52 target TFBS matrices showed a significantly higher multiplicity within the NCCRs of prototype JCVs from PML patients as compared with those of archetype JCVs from healthy individuals (Table 3). In the archetype JCVs from the urine of healthy individuals, each of these TFBS matrices were present in the NCCRs, except for matrix V$NFY.03, which was presented in duplicate. However, prototype JCVs detected in the CSF of PML patients had a higher number of TFBSs in the NCCRs than observed in archetype viruses. When TFBS matrices, often repeated in the NCCRs of prototype JCVs, were depicted in the NCCR of archetype JCV, these matrices were mainly distributed in regions C and E (Figure 4). Table 4 shows the gene ontology and expression profiles of transcription factors capable of binding to TFBS matrices with increased multiplicity in the rearranged NCCR of prototype JCVs. Genes encoding these transcription factors, except for lymphoid enhancer binding factor 1 and nuclear factor I (NFI) C, are expressed in various human tissues, including the brain. The gene-expression profile of SRY-box transcription factor 6 was not included in the Affymetrix microarray dataset used in this study but is reportedly ubiquitously expressed [67]. These data suggested that the limited number of TFBSs in regions C and E tend to overlap during the rearrangement of the NCCR sequences of prototype JCVs from a statistical standpoint, and that transcription factors capable of binding to these TFBSs are mostly ubiquitously expressed.

## 4. Discussion

In this study, the NCCR sequences of many JCV isolates were sorted and aligned, and their origins were checked individually. Additionally, we identified TFBSs by computer simulation and added these to the NCCR sequences as annotations. The resulting database can be used to visually display the location of TFBSs within the NCCRs of both archetype and prototype JCVs or extract a tabulated list of NCCRs that possess particular TFBSs. Although the TFBSs highly conserved in the archetype NCCRs were analyzed by data science or statistics in this work, the actual sequence list contained all identified TFBSs. This database could be used to compare not only the differences between archetype and prototype JCVs, but also the NCCR sequences of each virus type. For example, it may be applied to the analysis of TFBS among genotypes of archetype JCVs or to the statistical analysis of NCCR sequences of prototype JCVs among groups of PML patients divided by factors such as underlying disease and prognosis. Furthermore, the FASTA file dataset with TFBS annotations can be utilized by standard genetic analysis software without requiring advanced database-management skills, making it versatile for research on NCCRs.

We used the database created in this study to analyze features and trends in the patterns of TFBSs within the NCCR of the prototype JCV. A notable finding was that a limited number of TFBSs conserved in the NCCR sequences of archetype JCVs was often lost or overlapped in prototype viruses. Previous studies reported that the balance of promoter activities of early and late genes differs in the NCCRs of archetype and prototype JCVs [40,41]. It is likely that changes in TFBS patterns are associated with alterations in promoter activity. Interestingly, although we identified TFBS matrices with statistically distinct possession rates or multiplicities in the NCCRs of prototype JCVs as compared with the archetype viruses, these matrices were neither lost nor duplicated in some prototype JCVs. These observations imply that there is no universal pattern for TFBS deletion or multiplication during NCCR rearrangement, and that a vast number of TFBS combinations are being generated.

The TFBSs with lower possession rates in prototype JCVs were mainly distributed in regions D through F of the NCCR sequences of archetype viruses. There are numerous reports indicating that NCCRs of prototype JCVs frequently lack region D [68], which is consistent with the present findings. Additionally, we showed that regions E and F also tended to be deleted according to statistical analysis of the TFBS patterns in a large number of prototype JCVs. Moreover, analysis of data from the BioGPS database indicated that the transcription factors that bind to these TFBSs were expressed in diverse peripheral tissues, including sites of persistent or latent JCV infection. Notably, several transcription factors that bind to these TFBSs with reduced possession rates are highly expressed in CD34+ cells, which have been implicated in PML pathogenesis as sites of latent or persistent JCV infection [29]. By contrast, we did not observe specific and high-level expression of transcription factors, which bind to TFBSs with increased multiplicity, in CD34+ cells. Thus, several TFBSs lost upon NCCR rearrangement might control the promoter activity of archetype JCV in CD34+ cells during latent or persistent infection and are not required for lytic infection of prototype JCV in the brain.

The number of TFBSs prone to overlap was small as compared with those likely to be lost in prototype NCCRs. This feature can be attributed to the fact that it is not unusual to find prototype JCVs without duplicated sequences in NCCRs, and that the pattern of rearrangement is highly variable, causing few TFBSs to overlap in common. We found that the NCCR sequences in regions C and E of archetype JCV tended to be duplicated in the prototype viruses based on results from the count data model using TFBSs as landmarks. It is difficult to reveal the mutational patterns of rearranged NCCRs by simply comparing the alignment of nucleotide sequences; therefore, this result could provide valuable insights into the mutational trends of NCCRs. Additionally, examination of the gene-expression profiles of transcription factors capable of binding to the TFBSs (which are likely to overlap in the prototype JCV) revealed their expression in various tissues. Although these observations suggest the possibility that overlapping TFBSs in rearranged NCCRs facilitate JCV proliferation, this duplication event might not be involved in the tropism of this virus in the brain.

We expect that the database created in this study will serve as a convenient roadmap for future studies of NCCR functions and especially analyses of the activities of early and late promoters of JCV. It would be interesting to compare the results obtained from the TFBS database with the transcriptional activity of JCV promoters in vitro. For example, the TFBS matrix V$NF1.03, which tends to duplicate in the NCCRs of prototype JCV, is targeted by transcription factors belonging to the NFI family, which are reportedly involved in JCV gene expression and proliferation [61,69]. Another interesting example is that the TFBS matrix V$SPIB.01 for the Spi-B transcription factor, which reportedly plays an important role in JCV gene expression [61,70,71], is highly conserved in both archetype and prototype JCVs (possession rates: 100% and 96.7%, respectively) in the created database. Notably, the possession rate and multiplicity of V$SPIB.01 were not statistically significant in the archetype and prototype JCVs (*P* = 0.552 and *P* = 0.899, respectively), suggesting that TFBSs that are not lost or duplicated upon NCCR rearrangement play roles in JCV replication. As summarized in Supplementary Dataset S3, there are other TFBSs that are highly conserved in the NCCRs of both archetype and prototype JCVs. Among these TFBSs, it would be interesting to investigate the function of the TFBS matrix V$OLIG2.01, which is located at the downstream end of the forward strand of the NCCR. This TFBS includes a binding sequence for oligodendrocyte transcription factor 2 (OLIG2), which is expressed in oligodendrocytes and required for myelination [72]. It is speculated that OLIG2 might be involved in the glia-specific gene expression of JCV.

Furthermore, it would be interesting to analyze the promoter machinery of NCCRs in more detail by deleting these TFBSs individually or in combination based on their sequences and locations. MatInspector is available to perform TFBS simulations and use metadata based on a paid subscription. Under the terms of the license agreement of MatInspector, disclosing more than 10 DNA sequences of TFBSs in publications is legally restricted. However, in this study, Precigen Bioinformatics Germany GmbH, which does business as Genomatix and is the provider of this product, has kindly allowed us to disclose the actual DNA sequences presented in the Figures and Tables. Thus, we listed the DNA sequences of TFBSs within NCCR of representative CY strain of JCV and attached a digital file with the names and HGNC IDs of their corresponding transcription factors (Supplementary Dataset S4). In these TFBSs, the core sequences are common in the archetype and prototype JCVs. However, in the rearranged NCCRs of prototype JCVs, there are occasionally differences in DNA sequences other than the core of the TFBSs, which are defined as one of the same TFBS matrices. In such cases, it may be possible to find the TFBS by alignment with the annotated sequence list of NCCRs from many prototype JCVs, although a simple sequence search may not yield any hits. The dataset established in the present study will be made available for non-commercial purposes upon reasonable request. Additionally, for detailed analysis of TFBSs in the NCCR sequences, we recommend the use of MatInspector.

## 5. Conclusions

In conclusion, we described the creation and application of a database containing the NCCR sequences of archetype and prototype JCVs, as well as annotations of TFBSs within these NCCRs. Furthermore, statistical analyses clarified the observed alterations in TFBS patterns during NCCR rearrangements. We believe that the generated database and subsequent insights obtained in this study will contribute to further advances in the analysis of NCCR function and JCV pathogenicity.

## Figures and Tables

**Table 1:** Possession rates of TFBSs within the NCCRs of JCVs in the urine of healthy individuals and CSF of PML patients.

		Urine of Healthy Individuals (n = 49)		CSF of PML Patients (n = 91)		
Matrix Name	DNA Strand ^a^	JCV Isolates with Matrix ^b^	Possession Rate (%) ^c^	JCV Isolates with Matrix	Possession Rate (%)	*P*-Value ^d^
V$MIF1.01	FWD	47	95.9	19	20.9	<0.001
V$IRF1.01	FWD	47	95.9	20	22.0	<0.001
V$HOXC10.01	FWD	48	98.0	27	29.7	<0.001
V$PLAGL1.01	FWD	48	98.0	30	33.0	<0.001
V$PDX1.01	FWD	48	98.0	33	36.3	<0.001
V$CRX.01	FWD	48	98.0	34	37.4	<0.001
V$NKX61.01	FWD	48	98.0	34	37.4	<0.001
V$BRN5.03	FWD	48	98.0	36	39.6	<0.001
V$ZBTB3.01	FWD	47	95.9	59	64.8	<0.001
V$FOXJ3.01	FWD	48	98.0	62	68.1	<0.001
V$SPI1.02	FWD	49	100	80	87.9	0.008
V$IRF7.01	FWD	49	100	81	89.0	0.015
V$MZF1.02	FWD	49	100	81	89.0	0.015
V$CMYB.02	REV	47	95.9	23	25.3	<0.001
V$ROAZ.01	REV	48	98.0	28	30.8	<0.001
V$PLAGL1.01	REV	48	98.0	30	33.0	<0.001
V$ZNF232.01	REV	48	98.0	32	35.2	<0.001
V$ZBTB3.01	REV	47	95.9	32	35.2	<0.001
V$HOXC9.01	REV	48	98.0	33	36.3	<0.001
V$MEIS1.03	REV	48	98.0	33	36.3	<0.001
V$SMARCA3.02	REV	48	98.0	55	60.4	<0.001
V$PRDM4.01	REV	49	100	80	87.9	0.008

Abbreviations: CSF, cerebrospinal fluid; FWD, forward; JCV, JC virus; NCCR, non-coding control region; PML, progressive multifocal leukoencephalopathy; REV, reverse; TFBS, transcription factor binding site. ^a^ The direction of the DNA strand is mentioned in the Figure 2 legend. ^b^ The number of JCV isolates with the respective matrices for TFBSs in the NCCR. ^c^ The proportion of JCV isolates that possessed the respective matrices in each group. ^d^ The possession rates of 52 matrices between the two groups were analyzed using Fisher’s exact test and the Benjamini–Hochberg method. Matrices with statistically significant differences are shown.

**Table 2:** Profiles of TFBSs that are reduced in the NCCRs of JCVs in the CSF of PML patients.

		Transcription Factor ^a^			
Matrix Name	DNA Strand ^b^	HGNC ID	Symbol	Full Name	Gene Expression (>3 × Median) ^c^
V$MIF1.01 ^d^	FWD	4921/9982	HIVEP2/ RFX1	HIVEP zinc finger 2/ regulatory factor X1	Brain (cerebrum), Blood (T cells)/ NA (Ubiquitous)
V$IRF1.01	FWD	6116	IRF1	Interferon regulatory factor 1	Blood (Leukocytes), Bone marrow (CD34+ cells), Colon, Heart, Lung, Lymph node, Placenta, Small intestine, Thymus
V$HOXC10.01	FWD	5122	HOXC10	Homeobox C10	Kidney
V$PLAGL1.01	FWD REV	9046	PLAGL1	PLAG1-like zinc finger 1	Adrenal gland, Bone marrow (CD34+ cells), Colon, Pituitary gland, Placenta, Prostate, Retina, Small intestine, Smooth muscle, Uterus
V$PDX1.01	FWD	6107	PDX1	Pancreatic and duodenal homeobox 1	NA (Ubiquitous)
V$CRX.01	FWD	2383	CRX	Cone-rod homeobox	Pineal gland, Retina
V$NKX61.01	FWD	7839	NKX6-1	NK6 homeobox 1	NA (Ubiquitous)
V$BRN5.03	FWD	9224	POU6F1	POU class 6 homeobox 1	NA (Ubiquitous)
V$ZBTB3.01	FWD REV	22918	ZBTB3	Zinc finger and BTB domain containing 3	NA (Ubiquitous)
V$FOXJ3.01	FWD	29178	FOXJ3	Forkhead box J3	NA (Ubiquitous)
V$SPI1.02	FWD	11241	SPI1	Spi-1 proto-oncogene	Blood (Monocytes), Lung
V$IRF7.01	FWD	6122	IRF7	Interferon regulatory factor 7	Blood (Leukocytes), Bone marrow (CD34+ cells), Heart, Lung, Lymph node, Thymus, Tonsil
V$MZF1.02	FWD	13108	MZF1	Myeloid zinc finger 1	Blood (leukocytes), Blood vessel (endothelial cells), Bone marrow (CD34+ cells), Pineal gland, Prostate, Thyroid gland
V$CMYB.02	REV	7545	MYB	MYB proto-oncogene, transcription factor	Blood vessel (endothelial cells), Bone marrow (CD34+ cells), Thymus
V$ROAZ.01	REV	16762	ZNF423	Zinc finger protein 423	Brain (whole), Pineal gland, Retina, Small intestine, Uterus
V$ZNF232.01	REV	13026	ZNF232	Zinc finger protein 232	NA (Ubiquitous)
V$HOXC9.01	REV	5130	HOXC9	Homeobox C9	NA (Ubiquitous)
V$MEIS1.03	REV	7000	MEIS1	Meis homeobox 1	Adrenal gland, Bone marrow (CD34+ cells), Brain (cerebellum), Colon, Ovary, Salivary gland, Small intestine, Smooth muscle, Trachea, Uterus
V$SMARCA3.02	REV	11099	HLTF	Helicase like transcription factor	Blood (T cells and NK cells), Blood vessel (endothelial cells), Bone marrow (CD34+ cells), Pineal gland, Pituitary gland, Thyroid gland
V$PRDM4.01	REV	9348	PRDM4	PR/SET domain 4	Blood (B cells), Bone marrow (CD34+ cells), Pineal gland

Abbreviations: CD, cluster of differentiation; FWD, forward; HGNC, Human Genome Organization Gene Nomenclature Committee; ID, identification; JCV, JC virus; NA, not applicable; NCCR, non-coding control region; PML, progressive multifocal leukoencephalopathy; REV, reverse; TFBS, transcription factor binding site. ^a^ The gene ontology of transcription factors predicted to bind each sequence was confirmed using the HGNC database in accordance with the metadata of the matrices. ^b^ The direction of the DNA strands is mentioned in the Figure 2 legend. ^c^ Gene-expression profiles of transcription factors in human tissues and blood were obtained using BioGPS microarray data, and the sites with 3-fold higher expression levels relative to the median are indicated. ^d^ This matrix is defined as the sequence targeted by the HIVEP2–RFX1 complex.

**Table 3:** Multiplicity of TFBSs within the NCCRs of JCVs in the urine of healthy individuals and CSF of PML patients.

		Poisson Mean (95% CI) ^a^				
Matrix Name	DNA Strand ^b^	Urine of Healthy Individuals (*n* = 49)		CSF of PML Patients (*n* = 91)		*P*-Value ^c^
V$HIC1.01	FWD	1.04	[0.75, 1.33]	1.89	[1.59, 2.18]	<0.001
V$NF1.03	FWD	1.06	[0.77, 1.35]	1.93	[1.63, 2.23]	<0.001
V$NFY.03	FWD	2.00	[1.60, 2.40]	3.19	[2.81, 3.56]	<0.001
V$SOX6.01	FWD	1.02	[0.73, 1.31]	1.66	[1.39, 1.94]	0.001
V$LEF1.01	FWD	1.02	[0.73, 1.31]	1.63	[1.36, 1.90]	0.002
V$PAX9.02	REV	1.04	[0.75, 1.34]	1.89	[1.59, 2.18]	<0.001
V$PAX6.01	REV	1.04	[0.75, 1.34]	1.88	[1.58, 2.17]	<0.001

Abbreviations: CI, confidence interval; CSF, cerebrospinal fluid; FWD, forward; JCV, JC virus; NCCR, non-coding control region; PML, progressive multifocal leukoencephalopathy; REV, reverse; TFBS, transcription factor binding site. ^a^ The number of each matrix within the NCCRs of JCV isolates (multiplicity) was predicted by using the Poisson distribution for modeling count data. ^b^ The direction of the DNA strands is mentioned in the Figure 2 legend. ^c^ *P*-values were adjusted for multiple testing using the Benjamini–Hochberg method, and the matrices with statistically significant differences are shown.

**Table 4:** Profiles of TFBSs likely to multiply in the NCCRs of JCVs in the CSF of PML patients.

		Transcription Factor ^a^			
Matrix Name	DNA Strand ^b^	HGNC ID	Symbol	Full Name	Gene Expression (>3 × Median) ^c^
V$HIC1.01	FWD	4909	HIC1	HIC ZBTB transcriptional repressor 1	NA (Ubiquitous)
V$NF1.03	FWD	7784	NFIA	Nuclear factor I A	NA (Ubiquitous)
V$NF1.03	FWD	7785	NFIB	Nuclear factor I B	Brain (cerebrum, cerebellum, olfactory bulb), Colon, Ovary, Pancreatic islet, Prostate, Retina, Salivary gland, Skin, Small intestine, Smooth muscle, Tongue, Trachea, Uterus
V$NF1.03	FWD	7786	NFIC	Nuclear factor I C	Skeletal muscle
V$NF1.03	FWD	7788	NFIX	Nuclear factor I X	NA (Ubiquitous)
V$NFY.03	FWD	7804	NFYA	Nuclear transcription factor Y subunit alpha	NA (Ubiquitous)
V$SOX6.01	FWD	16421	SOX6	SRY-box transcription factor 6	NA ^d^
V$LEF1.01	FWD	6551	LEF1	Lymphoid enhancer binding factor 1	Blood (T cells), Thymus
V$PAX9.02	REV	8623	PAX9	Paired box 9	NA (Ubiquitous)
V$PAX6.01	REV	8620	PAX6	Paired box 6	Brain (cerebrum, cerebellum), Pancreatic islet, Pineal gland, Retina, Skeletal muscle

Abbreviations: CSF, cerebrospinal fluid; FWD, forward; HGNC, Human Genome Organization (HUGO) Gene Nomenclature Committee; ID, identification; JCV, JC virus; NA, not applicable; NCCR, non-coding control region; PML, progressive multifocal leukoencephalopathy; REV, reverse; TFBS, transcription factor binding site. ^a^ The gene ontology of transcription factors predicted to bind to each sequence was confirmed using the HGNC database according the metadata of the matrices. ^b^ The direction of the DNA strands is mentioned in the Figure 2 legend. ^c^ Gene-expression profiles of transcription factors in human tissues and blood were obtained using BioGPS microarray data, and the sites with 3-fold higher expression levels relative to the median are indicated. ^d^ The gene-expression profile for SOX6 was not included in the dataset.

## Data Availability

The analyzed datasets are available in the article and its Supplementary Materials, or are available from the corresponding author upon reasonable request.

## Supplementary Materials

The following are available online at www.mdpi.com/article/10.3390/v13112314/s1, Dataset S1: Summary of JCV DNA sequences containing the NCCR, Dataset S2: Specimens and health conditions of individuals in which JCV NCCR sequences were detected, Dataset S3: TFBS matrices in JCV NCCRs for which statistical analyses were performed, Dataset S4: Actual DNA sequences and counterparts of TFBSs in JCV NCCR, Figure S1: Histogram analysis of the possession rates of TFBS matrices in the archetype NCCRs.
