# Supplementary material for: Database and Statistical Analyses of Transcription Factor Binding Sites in the Non-Coding Control Region of JC Virus
Source: Viruses. 2021 Nov 19;13(11):2314. doi: 10.3390/v13112314 (PMC8620444; doi:10.3390/v13112314)
Supplement: Supplementary file 1 [file viruses-13-02314-s001.zip › Supplementary Figure S1.pdf]

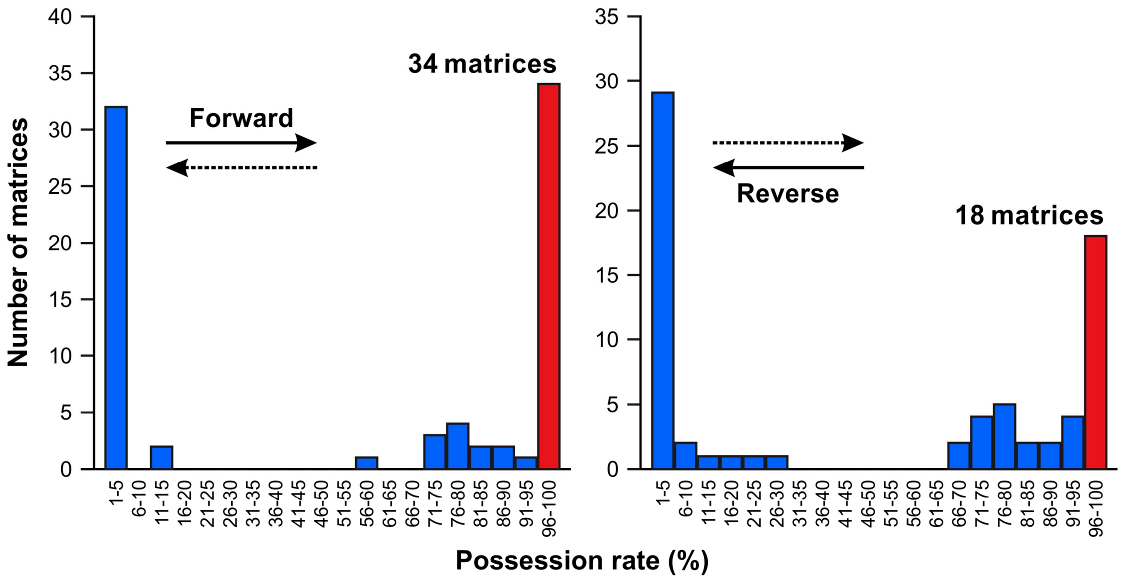

Figure S1. Histogram analysis of the possession rates of TFBS matrices in the archetype NCCRs. TFBSs in the NCCR sequences of 49 JCV isolates from the urine of healthy individuals were identified, and the numbers of each matrix in the entire virus population were counted. The results of analyses of TFBS matrices in the forward strand of the NCCR (left) and its complementary reverse strand (right). Red bars indicate the number of TFBS matrices possessed by > 95% of archetype JCV isolates.
